# Cross-cultural adaptation of the EMIC Stigma Scale for people with leprosy in Brazil

**DOI:** 10.11606/S1518-8787.2017051000167

**Published:** 2017-08-25

**Authors:** Fabiane Frota da Rocha Morgado, Erika Maria Kopp Xavier da Silveira, Anna Maria Sales, Lilian Pinheiro Rodrigues do Nascimento, Euzenir Nunes Sarno, José Augusto da Costa Nery, Aldair J Oliveira, Ximena Illarramendi

**Affiliations:** I Instituto de Educação. Departamento de Educação Física e Desportos. Universidade Federal Rural do Rio de Janeiro. Seropédica, RJ, Brasil; II Ambulatório Souza Araújo. Instituto Oswaldo Cruz. Rio de Janeiro, RJ, Brasil; III Laboratório de Hanseníase. Instituto Oswaldo Cruz. Rio de Janeiro, RJ, Brasil; IV Centro de Desenvolvimento Tecnológico em Saúde. Fundação Oswaldo Cruz. Rio de Janeiro, RJ, Brasil

**Keywords:** Leprosy, psychology, Social Stigma, Translations, Surveys and Questionnaires, utilization

## Abstract

**OBJECTIVE:**

Describe the process of cross-cultural adaptation of the “Explanatory Model Interview Catalog – Stigma Scale” for people affected by leprosy in Brazil.

**METHODS:**

After being authorized by the author of the scale to use it in the national context, we initiated the five steps process of cross-cultural adaptation: (1) translation, (2) synthesis meeting, (3) back-translation, (4) committee of experts and (5) pre-test. The internal consistency of the scale was evaluated using Cronbach’s alpha coefficient.

**RESULTS:**

The 15 items of the scale’s original version were translated into Brazilian Portuguese. The adapted scale showed evidence of a good understanding of its content, attested both by experts and members of the target population. Its internal consistency was 0.64.

**CONCLUSIONS:**

The adapted instrument shows satisfactory internal consistency. It may be useful in future studies that intend to provide broad situational analysis that supports solid public health programs with a focus on effective stigma reduction. In a later study, the construct’s validity, criterion, and reproducibility will be evaluated.

## RESUMO

**OBJETIVO:**

Descrever o processo de adaptação transcultural da “*Explanatory Model Interview Catalogue – Stigma Scale”* para pessoas afetadas por hanseníase no Brasil.

**MÉTODOS:**

Após a autorização do autor da escala para seu uso no contexto nacional, deu-se início aos cinco passos do processo de adaptação transcultural: (1) tradução, (2) reunião de síntese, (3) retrotradução, (4) comitê de peritos e (5) pré-teste. A consistência interna da escala foi avaliada utilizando o coeficiente alfa de Cronbach.

**RESULTADOS:**

Os 15 itens da versão original da escala foram traduzidos para a língua portuguesa do Brasil. A escala adaptada apresentou evidência de boa compreensão de seu conteúdo, atestada tanto por peritos como por membros da população alvo. Sua consistência interna foi de 0,64.

**CONCLUSÕES:**

O instrumento adaptado apresenta consistência interna satisfatória. Pode ser útil em estudos futuros que intencionem viabilizar ampla análise situacional que sustente programas sólidos de saúde pública com enfoque na efetiva redução de estigma. Em estudo ulterior será avaliada a validade de constructo, critério e reprodutibilidade.

## INTRODUCTION

Stigma can be understood as any negative attribute placed on a person or group of people^5^. It has usually been classified as a perceived or experienced stigma. The first is due to the ideation of negative attitudes or practices in society; the experienced stigma consists of the experience of discrimination from any member of society, family or friend^1^ ^,^ ^23^ ^,^ ^25^. It is, therefore, a complex construct that, when related to health, reflects disqualification of the individual or group of individuals identified with specific health problems^23^.

The stigma related to leprosy is considered a phenomenon of global repercussion that occurs in both endemic and non-endemic countries^21^. However, there are few publications on the subject, especially in Brazil. In addition to compromising disease control by delaying the search for diagnosis and treatment, the stigma can cause disorders in different areas of life^19^ ^-^ ^21^.

Considering the need to understand the characteristics of the problems related to stigma and its impact on health^11^ ^,^ ^24^, as well as the importance of evaluating interventions with a focus on its reduction^3^ ^,^ ^15^ ^,^ ^18^, it becomes fundamental to systematically evaluate this construct in the field of leprosy. Measuring it, however, is not a simple task^14^. Its multidimensional and dynamic characteristics^6^ make it more difficult to assess its magnitude or intensity^19^.

Despite this difficulty, some instruments have been used in the international scenario to measure stigma and its impact on patients with leprosy^14^ ^,^ ^21^. In Brazil, however, there is a shortage of measures for this purpose. The few studies that investigate the relationship between stigma and leprosy in the country are qualitative studies^9^ ^,^ ^10^ ^,^ ^13^ that do not use standardized and internationally validated instruments^4^ ^,^ ^7^, preventing comparison with other studies.

The stigma scale of the Explanatory Model Interview Catalogue (EMIC Stigma Scale)^22^, initially applied in India and adapted for different languages and cultures^16^ is considered simple and useful for assessing perceived and experienced stigma related to leprosy^20^ ^,^ ^25^ ^,^ ^23^. In addition, it has shown satisfactory values of reliability – internal consistency of 0.88 and test-retest reliability of 0.7 in a study carried out in India, with 806 people affected by leprosy^17^. Thus, the EMIC Stigma Scale could contribute to systematic investigations of stigma related to leprosy in Brazil.

However, to use this instrument in the country, it is necessary to subject it to a rigorous process of cross-cultural adaptation, due to the need to provide semantic, idiomatic, cultural and conceptual equivalence between the original version and the Portuguese version, and those are not provided by a general translation into the local language^2^. The methodological process of translation and cultural adaptation to prepare the EMIC Stigma Scale for use in Brazil is described in this study.

## METHODS

The transcultural adaptation process of the EMIC Stigma Scale was initiated after authorization by email of the original scale’s first author^22^. The five steps of the methodological procedure for transcultural adaptation suggested in the Guide of the American Association of Orthopedic Surgeons/Institute for Work and Health were used^2^: translation, synthesis, back-translation, committee of experts, and pre-test. The first four steps were carried out in collaboration with a team from the commercial translation company Flash Traduções, located in Rio de Janeiro.

The first stage consisted of the translation of the instrument from the English language into Portuguese. The translation was carried out by two independent translators, one of them native of an English-speaking country and the other Brazilian. Neither of the translators had knowledge about the subjects covered in the EMIC Stigma Scale. Both translators generated a translated version of the scale (coded as T1 and T2), including the title, Likert response options, and the 15 questions of the scale.

The second stage included the synthesis meeting, with the participation of 10 professionals, as specified: five researchers (four specialists in leprosy and one in stigma), the two translators who participated in the first stage, a synthesis judge and two assistant coordinators (these last three were Brazilian natives and specialists in the English language). In this step, a synthesis version (T12) was developed from the evaluation of translations T1 and T2. Each aspect of the translations was analyzed and discussed to provide a consensus between both versions to ensure equivalence. The synthesis process, consensuses, and dissent were documented for further analysis.

In the third stage, the instrument was back-translated from the Portuguese language into the original language, to analyze conceptual errors or inconsistencies in the translation process. Two other independent translators, one of them American with fluency in Portuguese and the other native Brazilian with expertise in English, did the back translation. Based on T12, two new versions were created, encoded as RT1 and RT2. Both back translators were unaware of the theoretical constructs explored in the EMIC Stigma Scale and had no contact with the scale in its original version.

In the fourth step, the experts committee meeting was held. All the material produced in the previous steps were analyzed. The team of 15 specialists was composed of four researchers, two methodologists with experience in the process of validation of scales, a language professional (native Brazilian and specialist in Languages) and other participants of previous stages – translators, judge of synthesis, back-translators, and assistant coordinators. The committee also had the participation of a person who had leprosy. The main objective of this committee was to produce a final version of the scale to be sent to the pre-test.

In advance of the experts committee, the experts received a form with the material produced in the previous steps and were oriented to evaluate the content of each topic of the scale (title, Likert response options, and 15 questions), their comprehension, clarity, relevance, and equivalences. The evaluation could be carried out with grades varying from -1 to 1: -1 = inadequate, needs to be rewritten; 0 = suitable; 1 = extremely suitable. The experts were free to modify or eliminate irrelevant or ambiguous items and suggest substitutes that best fit the target population. The data analysis was performed both qualitatively and by the analysis of the scores of the experts’ answers.

The fifth stage consisted of the pre-test with a convenience sample, to obtain information about the reactions of the respondents to each aspect of the final version of the scale, making it possible to identify and eliminate potential problems in the instrument. In total, three pre-tests were performed after approval by the Human Research Ethics Committee of the Instituto Oswaldo Cruz/FIOCRUZ (CAAE 50625615.9.0000.5248). We selected subjects who were being treated at the Ambulatório Souza Araújo (ASA) of FIOCRUZ, Rio de Janeiro, a reference unit certified by the Joint Commission International. Adult subjects of both genders, either released or in polychemotherapy treatment, were included. Cases with HIV (due to it being a stigmatizing disease), individuals with cognitive impairment, and patients in polychemotherapy were excluded for less than two months. All the patients signed the free and informed consent form applied by a staff researcher in an ASA’s private room. The interviews were recorded for further clarification, if necessary.

The content of the scale, the level of difficulty to answer it and the method of application (interview or self-application) were evaluated. To evaluate the content, the techniques suggested by Malhotra^8^ were used: “protocol analysis” (the patient responded to the questions while “thinking” aloud) and “interrogation” (the patient reproduced, in his/her words, his/her understanding of each item). Items that were not clear were noted. To assess the level of difficulty, participants answered two questions: “How difficult was it to answer the questions on this scale?” (0) = not at all; (1) = slightly; (2) = a lot; and “How tiring was it to answer these questions?” (0) = not tiring; (1) = slightly tiring; (2) = very tiring. In order to evaluate the possibility of using the EMIC Stigma Scale in a self-administered way, the participants answered by themselves seven randomly selected items (items 2, 4, 7, 8, 9, 10 and 12). They also pointed out which of the methods they considered best to respond by having the following options: (0) either way, both methods were comfortable; (1) I prefer the help of the researcher reading the questions; (2) I prefer to respond by myself. All the data obtained in the pre-tests were analyzed both qualitatively and by analyzing the scores of the participants’ responses.

In addition, the reliability of the scale was measured by the internal consistency (Cronbach’s alpha).

### Instruments

EMIC Stigma Scale – we used the original version of the scale developed in the English language^22^ and adapted in more recent studies^1^ ^,^ ^24^. The scale has 15 items with Likert scale response options, as follows: (3) “yes”; (2) “possibly”; (1) “uncertain”; (0) “no”. The answer “yes” indicates a strong and positive indication of stigma, therefore it was assigned the highest value (3 points); while “no” indicates a strong and negative response, having been assigned the lowest value (0 point). Question 2 has a reverse score. The higher the sum of the scores, the greater the indication of stigma.

*Forms developed for this study* – Forms were developed to guide the study’s steps.

## RESULTS AND DISCUSSION

In order to make an instrument fit to be used in another cultural context, a process of transcultural adaptation of high quality is fundamental. This allows us to maintain equivalence between the original and translated versions, which is essential for their characteristics and application to remain invariable in distinct cultural contexts^2^.

### First Step – Translation

The two versions (T1 and T2) translated from the EMIC Stigma Scale were congruent only in relation to the Likert response options, thus being translated: “yes, possibly, uncertain, no”. The title and the 15 items, although translated with similar meaning, presented at least a grammatical or semantic distinction between T1 and T2.

### Second Step – Synthesis Meeting

The expression “Explanatory model” of the scale title was synthesized as “*Modelo explicativo*” (T1), to the detriment of “*Modelo explanatório*” (T2). Although “*explanatório*” and “*explicativo*” are synonymous words, the second was chosen because it is more usual in our country. In both T1 and T2 versions, the expression “Catalogue” was translated as “*Catálogo*”; however, experts suggested the word “*Inventário*” since it is more commonly used for such instruments. Thus, the synthesis title was “*Escala de Estigma – Inventário de Entrevistas em Modelo Explicativo*”. The acronym was maintained similar to the original (EMIC-SS) to preserve an easy identification of the scale in cross-cultural studies.

Other questions were related to the EMIC-SS questions. Item 1 was different in versions T1 and T2. T1 was written in the negative: “*Se possível, você preferiria que as pessoas não soubessem sobre sua hanseníase*?” (If possible, would you rather people not know about your leprosy?). To avoid possible confusion generated by negative questions^8^, we chose T2, with a few adjustments: “*Se possível, você evitaria que as pessoas soubessem que você tem hanseníase*?” (If possible, would you prevent people from knowing that you have leprosy?).

In items 2, 3, 4, 5, 7, 9 and 10, the word “problem” had its synthetic version as “*doença*”. That choice was made because leprosy is classified as a disease, according to the World Health Organization. In addition, there was a consensus among experts that, if translated literally as a “*problema*”, the expression could assume a stigma-filled sense, interfering with the subject’s response.

Item 3, originally composed of two questions, was also the focus of discussion. Items with two sentences can generate ambiguity and double interpretation, which is why it was suggested to divide this item into two. However, this strategy would alter the calculation of the scores, making it impossible to compare with other studies that used the EMIC-SS. The item, therefore, remained as in the original. The term “self-respect” of this item, initially translated as “*autoestima*”, was changed to “*autorrespeito*”, in order to approximate the translation of the original meaning. Nevertheless, it was replaced by “*respeito próprio*”, to facilitate the understanding by the target population of the scale.

Other questions involved some specific words. For example, item 5 caused debate because of the translation of “community”. If translated as “*comunidade*”, in Brazil, and especially in Rio de Janeiro, the expression could be directly associated with “*favela*”. It was then necessary to look for equivalent expressions, such as “*meio social*”. In question 8, “condition” had its synthesis version translated as “*doença*”, already mentioned in other questions of the EMIC-SS, which was selected to maintain the congruence of the scale. Another example was “think less” of item 9, which was synthesized as “*desvalorizar*”, using colloquial language easier to understand. Other small adjustments were made to the T1 or T2 in order to achieve a version that would guarantee the equivalence of the translation.

### Third Step – Back-translation

The two back-translations (RT1 and RT2) did not point to significant conceptual errors or inconsistencies in the translation process but were useful to direct effective and consistent actions at the experts committee stage.

### Fourth Step – Experts Committee

In the expert’s assessment, the title, the Likert response options, and seven items were rated -1 by at least two experts. The other items on the scale received a score of 0 or 1 and, therefore, were not altered. It was also suggested that the scale be self-applied.

As for the title, a slight change was made to make it more fluent in the Portuguese language, resulting in: “*Escala de estigma do inventário de entrevistas em modelo explicativo*”. Regarding the Likert responses, although T1 and T2 agreed on the translation of “possibly”, the expression “*talvez*” was considered inadequate because it generated ambiguity. In the Portuguese language, “*talvez*” may suggest “maybe yes” or “maybe not”, making the expression different from its original meaning. Similarly, difficulties in translating “possibly” were found in a previous Indonesian study^14^. Given the impossibility of finding a tuned synonym in the local language – Bahasa Indonesia – the authors translated the expression as “*mungkin*”, equivalent to “maybe” in the original language. In this study, we opted for “*possivelmente sim*” to translate “possibly” as a way of trying to get closer to the original meaning.

Questions 3, 6, 8, 10, 11A, 11B and 12 were amended in order to facilitate their understanding. In item 3, the expression “Have you ever” was translated as “*Alguma situação*” instead of “*Já houve alguma situação*”. This change was made to approximate the meaning of the new sentence to that of its original English version. Likewise, in item 6, “*seu contato com pessoas a sua volta*” was replaced by “*o contato das pessoas com você*”. In item 8, we replaced “*Alguém se recusaria*” with “*Você acha que alguém se recusaria*”. In item 10, “*familiares*” were included to also include childless participants, since the item in its original version seems to assume that all participants have children. Items 11A, 11B, and 12 were amended to attempt to ensure cultural equivalence in order to include participants who have romantic and family constitutions different from traditional marriage. Considering that the three questions involve this theme, we translated the expressions “to get married”, “marriage”, and “to marry” as “*relacionamento amoroso*”. The expressions “Unmarried only” and “Married only” were respectively translated to “*Apenas para pessoas sem parceiro*” and “*Apenas para pessoas com parceiro*”.

The original scale and the changes described up to the fourth step (experts committee) can be seen in Box 1.

**Box 1:** Comparison of EMIC Stigma Scale in its original version, synthesis version and the version used in the first pre-test. Rio de Janeiro, Brazil, 2016.

Item	Original document	Synthesis of translations	Final version resulting from the Experts Committee and used in the first pre-test
01	If possible, would you prefer to keep people from knowing about your leprosy?	*Se possível, você evitaria que as pessoas soubessem que você tem hanseníase?*	*Se possível, você preferiria evitar que as pessoas soubessem que você tem ou teve hanseníase?*
02	Have you discussed this problem with the person you consider closest to you, the one whom you usually feel you can talk to most easily?	*Você já conversou sobre sua doença com a pessoa que você considera mais próxima de você, ou seja, aquela com quem você, geralmente, se sente mais à vontade para falar?*	*Você já conversou sobre a hanseníase com a pessoa que você considera mais próxima de você, ou seja, aquela com quem você, geralmente, se sente mais à vontade para falar?*
03	Do you think less of yourself because of this problem? Has it reduced your pride or self-respect?	*Você se considera inferir por causa desta doença? Ela diminui seu orgulho ou respeito próprio?*	*Você se considera inferior por causa desta doença? Ela diminui seu orgulho ou respeito próprio?*
04	Have you ever been made to feel ashamed or embarrassed because of this problem?	*Já houve alguma situação ou alguém que fez você se sentir envergonhado ou constrangido por causa desta doença?*	*Alguma situação já fez você se sentir envergonhado ou constrangido por causa desta doença?*
05	Do your neighbors, colleagues or others in your community have less respect for you because of this problem?	*Seus vizinhos, colegas ou outras pessoas de seu meio social demonstram menos respeito por você por causa desta doença?*	*Seus vizinhos, colegas ou outras pessoas de seu meio social demonstram menos respeito por você por causa desta doença?*
06	Do you think that contact with you might have any bad effects on others around you even after you have been treated?	*Você acha que seu contato com pessoas a sua volta poderia ter algum efeito ruim para elas, mesmo após seu tratamento?*	*Você acha que o contato das pessoas com você poderia ter algum efeito ruim para elas, mesmo após seu tratamento?*
07	Do you feel others have avoided you because of this problem?	*Você sente que as pessoas têm te evitado por causa desta doença?*	*Você sente que as pessoas têm te evitado por causa desta doença?*
08	Would some people refuse to visit your home because of this condition even after you have been treated?	*Alguém se recusaria a ir a sua casa por causa de sua doença, mesmo após seu tratamento?*	*Você acha que alguém se recusaria a ir a sua casa por causa de sua doença, mesmo após seu tratamento?*
09	If they knew about it would your neighbors, colleagues or others in your community think less of your family because of your problem?	*Se seus vizinhos, colegas ou outras pessoas de seu meio social soubessem sobre sua doença, eles poderiam desvalorizar sua família por isso?*	*Se seus vizinhos, colegas ou outras pessoas de seu meio social soubessem sobre sua doença, eles poderiam desvalorizar sua família por isso?*
10	Do you feel that your problem might cause social problems for your children in the community?	*Você sente que sua doença poderia trazer problemas para seus filhos em seu meio social?*	*Você sente que sua doença poderia trazer problemas para a vida social de seus filhos ou familiares?*
11A	Do you feel that this disease has caused, or will cause, problems for you to get married? (Unmarried only)	*Você sente que esta doença causou ou irá causar dificuldades para você se casar? (Apenas para solteiros)*	*Você sente que esta doença causou ou causará dificuldades para você ter um relacionamento amoroso? (Apenas para pessoas sem parceiro)*
11B	Do you feel that this disease has caused problems in your marriage? (Married only)	*Você sente que esta doença tem causado problemas em seu casamento? (Apenas para casados)*	*Você sente que esta doença causa problemas em seu relacionamento amoroso? (Apenas para pessoas com parceiro)*
12	Do you feel that this disease makes it difficult for someone else in your family to marry?	*Você sente que esta doença dificulta que alguém de sua família se case?*	*Você sente que esta doença dificulta que alguém de sua família tenha um relacionamento amoroso?*
13	Have you been asked to stay away from work or social groups?	*Já pediram a você para ficar afastado de seu trabalho ou de grupos sociais?*	*Já pediram a você para ficar afastado de seu trabalho ou de grupos sociais?*
14	Have you decided on your own to stay away from work or social group?	*Você decidiu por conta própria ficar afastado de seu trabalho ou de grupos sociais?*	*Você decidiu por conta própria ficar afastado de seu trabalho ou de grupos sociais?*
15	Because of leprosy, do people think you also have other health problems?	*Por causa da hanseníase, as pessoas acham que você também tem outros problemas de saúde?*	*Por causa da hanseníase, as pessoas acham que você também tem outros problemas de saúde?*
Title	Explanatory Model Interview Catalogue Stigma Scale	*Escala de Estigma do Inventário de Entrevistas em Modelo Explicativo*	*Escala de Estigma do Inventário de Entrevistas em Modelo Explicativo*
Answers	Yes, possibly, uncertain, no	*Sim, talvez, não tenho certeza, não*	*Sim, possivelmente sim, não tenho certeza, não*

### Fifth Step – Pre-Test

The three pre-tests were performed following recommendations^8^ of between 5 and 10 participants for each pre-test, depending on the information saturation point. Each interview lasted approximately 50 minutes. We added text with instructions for the participants, which had to be adjusted along the pre-tests. The questions that presented problems or difficulties of understanding by the interviewees were changed for the subsequent pre-test.

The initial sample consisted of 29 subjects, but two refused to participate due to lack of time and three participants were excluded due to inability to understand the Likert scale. The final sample consisted of 24 participants, with a mean age of 44.8 years (SD = 13.25); the majority were male (54.2%) and natural of Rio de Janeiro (75.0%) (Table). Other participants were from Bahia (9%), Pernambuco, São Paulo, Maranhão and Rio Grande do Norte (4% each). Regarding education, the highest proportion (46%) reported incomplete primary education, but 17% had higher education. Regarding leprosy, the majority (83%) had been classified as multibacillary (with acid-alcohol resistant bacilli in a lymph node or skin or nerve biopsy), of which 14 (70%) had a lepromatous form. Of the total participants, 72% had been discharged after being cured.

**Table:** Sociodemographic characteristics of participants. Rio de Janeiro, Brazil, 2016.

Variable	Pre-test 1	Pre-test 2	Pre-test 3
Total number of participants	7	9	8
Gender (male/female)	5/2	5/4	3/5
Average age (years)	48,2 (SD = 15,7)	44,7 (SD = 12,5)	41,7 (SD = 12,6)
Minimum-maximum age (years)	26–64	27–66	29–64
Operational classification (PL/ML)	1/6	2/7	2/6
Married (yes/no)	5/2	6/3	4/4
Education			
ES (c/inc)	1/4	1/3	1/4
HS (c/inc)	0/1	2/1	1/1
HE (c/inc)	1/0	2/0	1/0

PL: paucibacillary leprosy (negative sputum smear microscopy); ML: multibacillary leprosy (positive smear microscopy); ES: elementary school; HS: high school; HE: higher education; c: complete; inc: incomplete; SD: standard deviation

As the main outcome of the first pre-test, we observed that some participants would change their response if their acquaintances (such as neighbors, friends, and co-workers) knew about their illness. Lustosa et al.^7^ emphasize that, in Brazil, there is a strong tendency for patients to hide that they are affected by leprosy. This issue directly impacts participants’ responses, since it limits their experiences with stigma. In order to respond to the EMIC-SS, the participant had to hypothesize the situation, but this capacity varies widely between participants and may compromise the validity of the scale. In this sense, future studies need to create or adapt scales that measure stigma experienced considering the frequent omissions by the patients.

In relation to completion of the scale, it was considered “not tiring” by all participants, a relevant result insofar as this instrument is possibly useful in future investigations that evaluate other associated constructs. On the other hand, four participants reported having no difficulty in responding to the scale, while three reported a slight difficulty and reported difficulty in understanding some items (2, 4, 6, 8 and 12).

Regarding the method of application, although the EMIC-SS was created in the interview model, a previous study used the scale in a self-administered way, with the justification that the interview model is costly and difficult to apply in large samples^12^. Since the data collection method can influence the interviewee during the stigma evaluation^14^, the researcher must be careful not to cause any discomfort to the person affected by leprosy, and training of the interviewers is therefore fundamental. Concerned with these issues, and after the recommendations of the experts who were especially concerned with the autonomy of the participants at the moment of data collection, the self-applied method was tested. Nevertheless, the traditional interview model was considered the most adequate by the participants (n = 3 preferred, n = 3 indifferent). Thus, the subsequent pre-tests were applied in an interview model.

In the second pre-test, most participants (n = 6) reported no difficulty answering the questions. The three participants who reported difficulty when answering, highlighted difficulties in understanding questions 2, 3, 13, 14 and 15. In particular, question 3, consisting of two questions, had already been identified as problematic by the experts committee. In the same vein, one of the participants verbalized that he would have an answer to the first question and another answer to the second question. In order to reduce this problem, the two questions were joined using the expression “*ou seja*”, resulting in “*Você se considera inferior por causa desta doença, ou seja, ela diminui seu orgulho ou respeito próprio*?”

In the third pre-test, although two participants still considered the scale “a bit” difficult to answer, the content of the questions was understood by all participants. Therefore, no other pre-test was considered necessary.

### Reliability

The mean of the total EMIC-SS score was 12.4 (SD = 6.0), with a range between 1 and 23. The internal consistency of the scale was 0.64, considered satisfactory for the present exploratory study^8^. Mean score similar to that found here was reported in a previous study^17^ – 13.8 (SD = 12), variation = 0 to 54. The internal consistency of the same previous study was higher (0.88) than our finding, possibly justified by the high number of participants (n = 806). The reliability of the translation of the EMIC-SS will be evaluated in a larger sample than the one used in this study.

The final version of the adapted scale can be seen in Box 2.

**Box 2:** Explanatory Model Interview Catalogue Stigma Scale (EMIC-SS).

n	Item
1	If possible, would you prefer to keep people from knowing about your leprosy? [*Se possível, você preferiria evitar que as pessoas soubessem que você tem ou teve hanseníase?*]
2	Have you discussed this problem with the person you consider closest to you, the one whom you usually feel you can talk to most easily? [*Você já conversou sobre sua hanseníase com a pessoa que você considera mais próxima de você, ou seja, aquela com quem você se sente mais à vontade para falar?*]
3	Do you think less of yourself because of this problem? Has it reduced your pride or self-respect? [Você se considera inferior por causa desta doença, ou seja, ela diminui seu orgulho ou respeito próprio?]
4	Have you ever been made to feel ashamed or embarrassed because of this problem? [*Já houve alguma situação que fez você se sentir envergonhado(a) ou constrangido(a) por causa desta doença?*]
5	Do your neighbors, colleagues or others in your community have less respect for you because of this problem? [*Seus vizinhos, colegas ou outras pessoas de seu meio social demonstram menos respeito por você por causa desta doença?*]
6	Do you think that contact with you might have any bad effects on others around you even after you have been treated? [*Você acha que o contato das pessoas com você poderia ter algum efeito prejudicial para elas, mesmo após seu tratamento?*]
7	Do you feel others have avoided you because of this problem ? [*Você sente que as pessoas têm te evitado por causa desta doença?*]
8	Would some people refuse to visit your home because of this condition even after you have been treated? [Alguém se recusaria a ir a sua casa por causa de sua doença, mesmo após seu tratamento?]
9	If they knew about it would your neighbors, colleagues or others in your community think less of your family because of your problem? [*Se seus vizinhos, colegas ou outras pessoas de seu meio social soubessem sobre sua doença, eles poderiam desvalorizar sua família por isso?*]
10	Do you feel that your problem might cause social problems for your children in the community? [*Você sente que sua doença poderia trazer problemas para a vida social de seus filhos ou familiares?*]
11A	Do you feel that this disease has caused, or will cause, problems for you to get married? (Unmarried only) [*Você sente que esta doença causou ou causará dificuldades para você ter um relacionamento amoroso? (Apenas para pessoas sem parceiro(a))*]
11B	Do you feel that this disease has caused problems in your marriage? (Married only) [*Você sente que esta doença causa problemas em seu relacionamento amoroso? (Apenas para pessoas com parceiro(a))*]
12	Do you feel that this disease makes it difficult for someone else in your family to marry? [*Você sente que esta doença dificulta que alguém de sua família tenha um relacionamento amoroso com outra pessoa?*]
13	Have you been asked to stay away from work or social groups? [*Já pediram a você para ficar afastado(a) de seu trabalho ou de grupos sociais por ter hanseníase?*]
14	Have you decided on your own to stay away from work or social group? [*Você decidiu por conta própria ficar afastado(a) de seu trabalho ou de grupos sociais por causa da doença?*]
15	Because of leprosy, do people think you also have other health problems? [*As pessoas acham que por você ter hanseníase também tem outros problemas de saúde?*]

Note: The Likert response options are defined as follows: “yes, possibly, uncertain, no”. The instructions to the participants are thus defined: “Dear [PARTICIPANT NAME], as a result of leprosy, its possible intercurrences and its different symptoms, we would like to know how this has affected the way you behave with others and how other people behave with you. There is no right or wrong answer. Take your time, reflect and answer what is true for you. You can ask the interviewer to repeat the questions as many times as necessary. Remember that all questions refer to your getting sick with leprosy”. [*As opções Likert de resposta são assim definidas: “sim, possivelmente sim, não tenho certeza, não”; As instruções aos participantes são assim definidas: “Prezado(a) [NOME DO PARTICIPANTE], como resultado da hanseníase, de suas possíveis intercorrências e de seus diferentes sintomas, nós gostaríamos de saber como isso tem afetado a maneira como você se comporta com os outros e como outras pessoas se comportam com você. Não há resposta certa ou errada. Tome seu tempo, reflita e responda o que é verdade para você. Pode pedir para repetir as perguntas tantas vezes quantas forem necessárias. Lembre-se que todas as perguntas se referem ao seu adoecer com hanseníase.*]

Two main limitations of this study must be highlighted. The first relates to the sample for convenience, which may limit the generalization potential of the results. However, considering that cross-cultural adaptation is a qualitative study, its intention is not to generalize, but to meet the equivalences required in this methodological process. In addition, although our sample was collected at a specialized center in the city of Rio de Janeiro, it was possible to verify that there was sample heterogeneity both in relation to place of birth, education level, gender, and age, among other aspects.

The second limitation refers to the interrogation technique, recommended by Malhotra^8^, used in the pre-test. Although commonly employed, this technique presented regular results in this study, since most of the patients had great difficulty reproducing with their own words what they understood about the item. We associated this result with the cognitive limitation of some participants, since in the first part of the pre-test – protocol analysis – it was clearly observed that the participants understood the questions. Future studies need to evaluate the effective applicability of the interrogation during the pre-test.

## CONCLUSIONS

The present study described the process of cross-cultural adaptation of the EMIC-SS for people affected by leprosy in Brazil. The adapted scale shows satisfactory internal consistency. It is of special value for future studies to enable the understanding of the situation of people affected by leprosy and to monitor, evaluate, and compare different intervention strategies and public health programs with a focus on effective stigma reduction. However, the use of scales in investigations in Brazil is still limited, and it is fundamental to carry out future studies that investigate its construct validity, criteria, and reproducibility in the national context.
